# Folded Sheets as a Universal Material for Shaping Transformed Shell Roofs

**DOI:** 10.3390/ma14082051

**Published:** 2021-04-19

**Authors:** Jacek Abramczyk

**Affiliations:** Department of Architectural Design and Engineering Graphics, Rzeszow University of Technology, Al. Powstańców Warszawy 12, 35-959 Rzeszów, Poland; jacabram@prz.edu.pl

**Keywords:** thin-walled folded sheets, elastic shape transformations, corrugated roof shells, smooth ruled surface models, constitutive mechanical relations, computational simulations, free-form buildings

## Abstract

This article provides a novel insight into specific properties of flat folded sheets transformed elastically into building roof shells. Elastic twist transformations of the sheets resulting from the arrangement of the sheets on two skew roof directrices cause changes in the geometric and mechanical sheet properties of the roof shell sheeting composed of these sheets. Regular smooth-ruled surfaces and their characteristic lines are used in the analysis of changes in the geometric properties. In the analysis of the mechanical changes, the constitutive relations and complex state of stresses are considered. The analysis is carried out on the basis of the results of the experimental tests and FEM computer simulations. They have led to the development of such a method of shaping of the effectively transformed folded covers that ensures the initial effort of each shell fold to be the smallest possible.

## 1. Introduction

**Figure 1:**
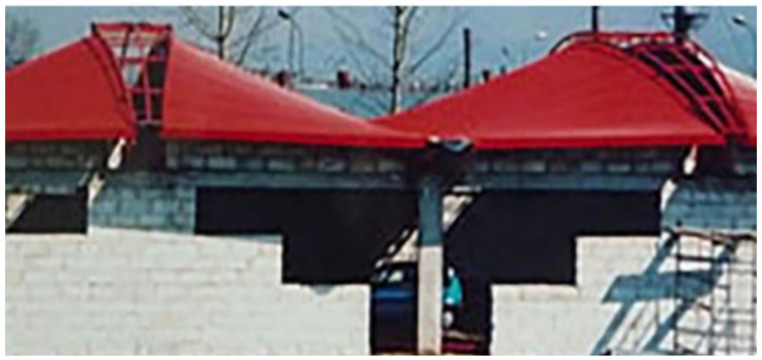
Transformed roof shells characterized by the top layer composed of visible sheets.

**Figure 2:**
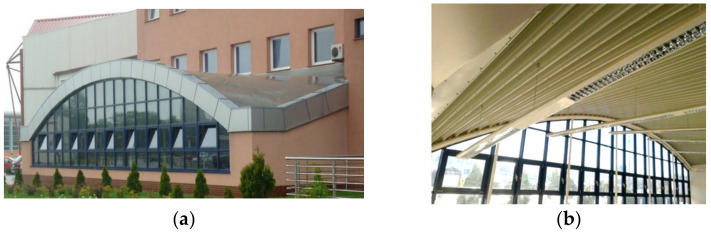
Transformed roof shell with bottom layer composed of folded sheets: (**a**) an outside view; (**b**) an inside view.

The main purpose of folding smooth thin steel sheets is to give them the structural properties needed to carry characteristic roof loads. The profiled sheets fulfil two essential functions. They take part in the load bearing and have protective functions; for example, against atmospheric influences [1]. Additionally, the sheets can form an outer roof layer, performing aesthetic functions and emphasizing the architectural qualities of the roof and the entire building (Figure 1). However, due to the durability of the external paint coatings guaranteed by producers for 50 years only, the folded sheets most often constitute the bottom load-bearing layer, supporting thermal insulation and the insulation foil of the roof (Figure 2a,b).

The aim of the elastic shape transformations of the sheets folded in one direction is to increase the visual attractiveness of roofs and entire buildings. On the other hand, the transformations make it possible to reduce the roof erection costs, inter alia, by using the sheets as lost shell formwork. Apart from the above essential advantages, there is a serious disadvantage of the shape transformations. The transformations induce great values of pre-stresses and large deformations of the thin walls of the sheets if they are not reduced by means of an appropriate calculation method, a way of loading and a technique of fixing the sheets to the roof construction based on the specific geometric and mechanical orthotropic properties of the folded sheets.

## 2. Critical Analysis of the Present Knowledge

**Figure 3:**
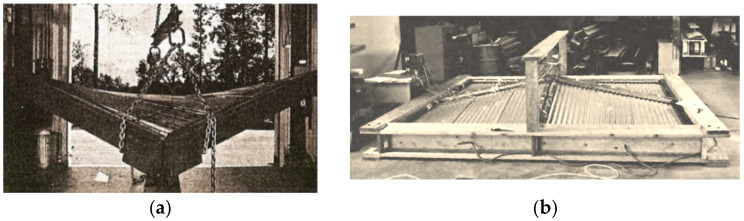
Two symmetric experimental hyperbolic paraboloid shells: (**a**) a complete shell; (**b**) an umbrella structure of four quarters.

Nominally, plane thin-walled folded steel sheets of open profiles are transformed into shell shapes to obtain doubly-curved covers. They are most often modeled with two-dimensional regular ruled surfaces. The behavior of a central sector of a folded steel hyperbolic paraboloid stiffened with a circumferential frame was studied by McDermott [2], Experimental studies of umbrella roofs composed of transformed hyperbolic parabolic quarters were first undertaken by Nilson [3] (Figure 3a). He proved that two-layer fold sheeting transformed elastically into a central sector of a hyperbolic paraboloid is more economical than the corresponding reinforced concrete hyperbolic paraboloid shell.

The most comprehensive research on two-layer transformed hyperbolic parabolic shells was carried out by Winter [4] at Cornell University. He studied a greater variety of the sheet profiles and dimensions of two-layer central sectors and compositions of quarters of hyperbolic paraboloid roofs. The obtained results were consistent with those presented by Nilsen. Various types and methods of determining ruled surfaces, including hyperbolic paraboloids, are described by Carmo [5] and Grey [6].

These shells were subjected to forced shape transformations, causing relatively big initial stresses. The negative impact of the forced transformations was enhanced by the bolt joints between two orthogonal layers arranged over the whole area of each transformed shell unit and the frame stiffened the entire complex shell. As a result, only shallow hyperbolic paraboloid shells called hypars could be created in that way [7].

Parker examined roof structures composed of four folded hyperbolic paraboloid quarters (Figure 3b). These units are also made up of two layers of sheets located orthogonally in two directions. He studied the changes in stiffness and stresses of the units [8]. Muscat primarily focused on the critical loads and stability of the corrugated shell sheeting [9]. Banavalkar performed an analysis related to static-strength work of the hypars [10].

**Figure 4:**
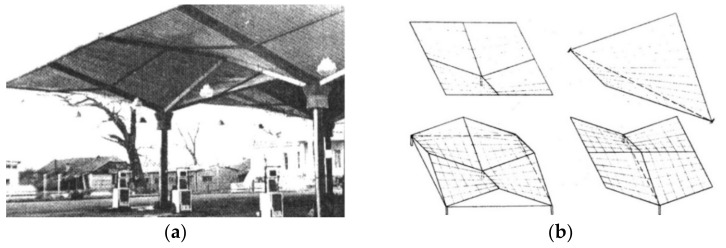
Symmetrically arranged hyperbolic paraboloid units: (**a**) an erected corrugated umbrella shed; (**b**) various configurations of umbrella shell structures.

Gioncu and Petcu [11] studied the principle of the work of the hyperbolic paraboloid shells using the traditional analytical analysis of strength and critical load. They invented a novel HYPBUCK computer program. They analyzed umbrella shell sheeting composed of four symmetrical hyperbolic paraboloid units in various configurations (Figure 4a,b).

Analogous studies related to the static strength work of single and complex hyperbolic paraboloid shells made up of flat folded sheets of different profiles were carried out by Egger et al. [12]. They performed tests, a conventional analysis, and analytical calculations of strength and critical loads.

**Figure 5:**
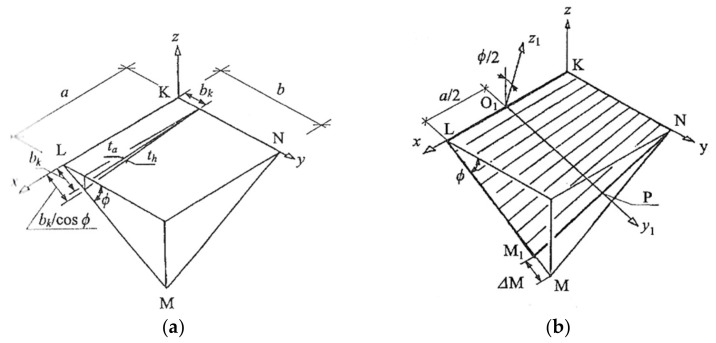
Adjustment of the longitudinal fold’s axes to a few selected rulings of one-fourth of a right hyperbolic paraboloid, forcing significant change in the width of the transverse fold’s ends: (**a**) forced change of transverse edge of individual transformed sheet, (**b**) forced change of transverse edge of complete transformed shell.

The above-mentioned researchers adopted the following traditional concept of geometric shaping of the transformed shells. At the beginning, they assumed the shape of a spatial quadrangle modeling the edge line of the designed transformed shell. Then they adopted two opposite lines; for example, LM and KL (see Figure 5a,b), as the directrices of the transformed sheeting. These directrices are linear elements of the roof construction supporting the designed shell along its edge line passing transversely to the fold’s direction. The segments KL and MN model the longitudinal edges of the border folds of the sheeting. After unfolding the subsequent sheets on the skew directrices, all folds try to adjust their shape to the mutual position of the directrices. Thus, the folds change the shape of their cross-sections, including the width, along their length depending on the supporting conditions that change along the length of each directrix.

Since the directrix LM is longer than the directrix KN in each right hyperbolic paraboloid quarter, the subsequent sheets are spread only over the section LM_1_ of the directrix LM. To cover the empty triangle MM_1_N with the transformed sheeting, its transverse ends passing along the directrices must be stretched, which causes additional stresses in relation to the ones caused by the free unfolding of the sheets on the skew directrices.

If b_k_ is the length of one transverse end of a sheet supported by the directrix KN (Figure 5a), the opposite end must be extended to the length b_k_/cos (ϕ) so that the entire directrix LM could be covered with the transverse ends of all subsequent sheets of the designed roof shell. The angle f between the directrices KN and LM is the total twist angle of the outside sheet containing the edge KL, if the directrices are rulings of a quadrant right hyperbolic-paraboloid sector.

The ϕ total twist angles of all subsequent folds of the shell modeled with the sector are different from themselves, which results from the changes in the inclination angles of the longitudinal edges of these folds to the directrices. The angle between the edges KL and KN is always right for the case of a quadrant sector of a right hyperbolic-paraboloid. The same is true for the angle between KL and LM. On the other hand, the angle between KN and NM or between NL and ML is different from right. Thus, the total twist angle of each shell fold, denoted in Figure 5 as ϕ, is congruent with the angle of the inclination of two planes defined by each of the directrices and a longitudinal edge of the fold.

The unit twist angle of this sheet is the quotient of the ϕ total twist angle and the a length of the sheet expressed in meters. The length of each transverse end of a transformed sheet depends on the profile type, length and degree of twisting of its subsequent folds.

The adjustment of all longitudinal shell fold’s longitudinal edges to the adopted finite number of rulings of the designed hyperbolic paraboloid quarters imposes a significant change in the width Δ*M* of the transverse fold’s ends passing along each roof directrix *LM* and important initial stresses [13] (Figure 5b). Therefore, the conventional methods drastically limit the variety of the designed transformed folded shell forms to central sectors of right hyperbolic paraboloids [13] and their quarters [14,15]. Therefore, the shells designed by means of these methods enforce unjustified additional stresses resulting from the need for the adjustment of the shell fold’s longitudinal axes to the locations of a selected few rulings and the length *LM* of the adopted directrix. The additional forces cause a reduction of the searched shell forms to shallow hypars.

Davis and Bryan [16] described the most important geometrical and mechanical characteristics of thin-walled flat and transformed shell folds. They showed a complete way of analyzing and designing various shells and structures made up of two-layer corrugated sheets located in two orthogonal directions. Two most important general conclusions given by these authors, and regarding the transformed roof shells, are as follows. They found that, theoretically, it is possible to shape many different configurations of the transformed folded shell sheeting. Practically, it is possible to build only cylindrical and a few hyperbolic paraboloid types of the transformed roof shells due to the available technology and the technique of the assembly of the nominally plane folded sheets.

Therefore, the use of the conventional design methods [1,7,11,17] in shaping such transformed two-layer shell roofs is ineffective. The methods usually result in high levels of transverse tensile normal stresses, local buckling and distortion of thin-walled walls. On the basis of the results achieved by the above-mentioned researchers, it can be stated that the assembly of each designed shell sheeting onto skew roof directrices is often impossible because of the plasticity of the fold’s edges between the flanges and webs or transverse ends of the flanges of the most transformed shell folds.

In the 1990s, Reichhart elaborated a new method for shaping deformed corrugated steel shells [13]. The algorithm of this method results from the specific orthotropic geometric and mechanical properties of elastically transformed folded sheeting [18]. Its main advantage is that the initial stresses are the smallest possible and the freedom of the transversal width increments of all shell folds is assured. His method relies on modeling the subsequent folds by means of sectors of right hyperbolic paraboloids, which enables one to reduce the initial effort of all shell folds belonging to deep, medium or even shallow right ruled surfaces. However, the Reichhart’s method is only accurate if the fold’s directions are perpendicular to the contraction of the designed transformed shell or very close to those. This is caused by the fact that he did not introduce a condition related to the location of the contraction of the transformed shell folds.

**Figure 6:**
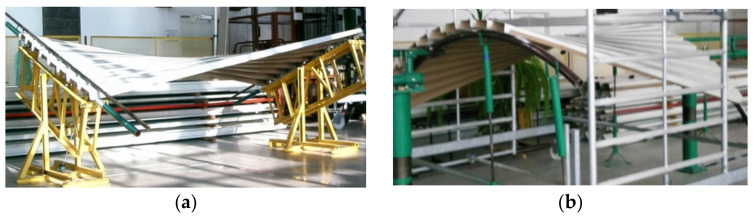
Experimental transformed corrugated shells supported by: (**a**) straight directrices, (**b**) curved directrices.

**Figure 7:**
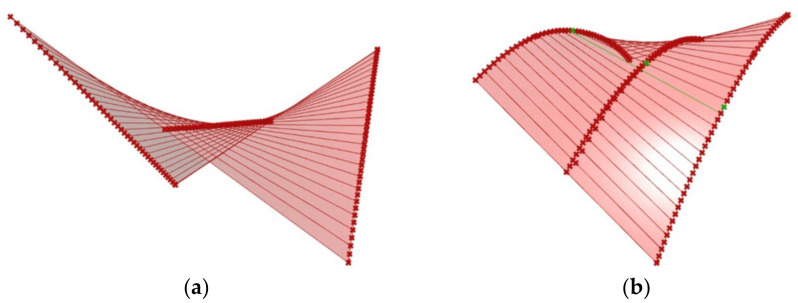
Smooth models of two transformed shells having straight and curvilinear lines of striction, shaped in the Rhino/Grasshopper program and defined with: (**a**) straight directrices; and (**b**) curved line of striction and directrices.

Abramczyk [19] invented a condition requiring the contraction of the entire corrugated shell sheeting to pass halfway along the length of each its fold (Figure 6a,b). He utilized the lines of striction of various warped surfaces (Figure 7a,b) to obtain smooth models of shell folds characterized by the effectiveness of their shape transformations [20]. The Abramczyk’s method also relates to calculations of the respective surface areas modeling compressing and stretching zones on the transformed folds [21]. The condition was defined on the basis of his analysis related to the results of the experimental tests [22] and computer simulations [21,23].

Samyn [24] elaborated a method of shaping two-layer transformed folded aluminum shells and their structures. He has also explored the possibility of using PVC trapezoidal plastic sheets for transformed coverings [25].

Transformed folded sheets can increase the visual attractiveness of the roof when viewed from the outside (Figure 1) or the inside (Figure 2b). Most often, however, in order to increase the durability of the roof, the sheeting is used as a bottom load-bearing layer supporting thermal insulation and insulating foil (Figure 2a).

As a result of joining the sheets with their longitudinal edges and supporting them on different skew directrices, unconventional coatings are formed [18,19]. They have a rather small span equal to the length of the shell folds and a contraction passing transversally in relation to the fold’s directions. Transformed shells of medium and large spans may be considered at a relatively small level of accuracy, where many individual shells with similar geometric properties are regularly distributed in the three-dimensional Euclidean space [26].

**Figure 8:**
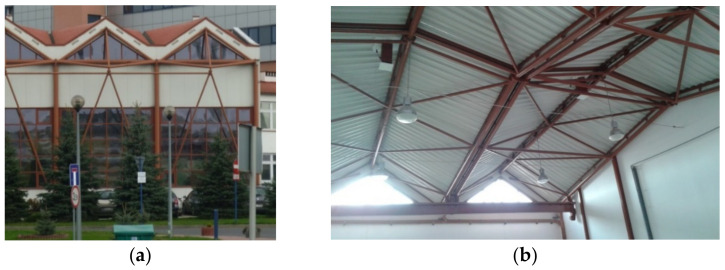
A roof shell structure composed of many transformed fold shell strips: (**a**) view from the outside; (**b**) view from the inside.

Reichhart also elaborated a method for arranging many complete corrugated shells on a horizontal or oblique plane [18] to achieve various continuous ribbed shell roof structures (Figure 8). Each corrugated shell sheeting designed by Reichhart is stiffened with a spatial framework, intermediate girders and oblique bracings [19].

**Figure 9:**
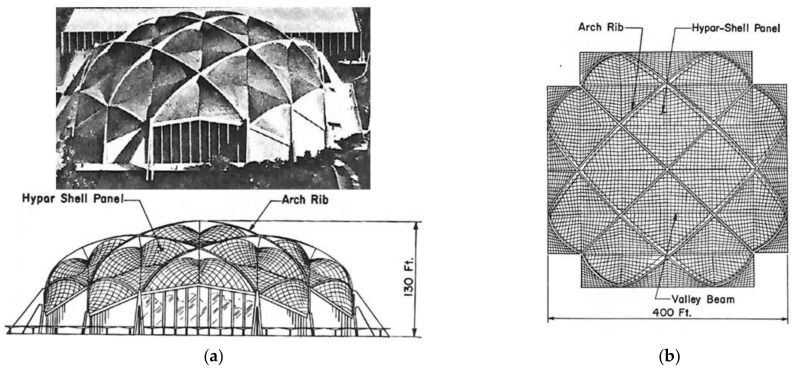
The Field House structure invented by Biswas and Iffland: (**a**) concept and elevation; (**b**) plan.

A method for shaping regular ribbed roof structures composed of many identical ruled sectors made up of transformed folded steel sheets and arranged on one sphere was defined by Biswas and Iffland [27]. In the first concept, shown in Figure 9a,b, they proposed triangular revolved hyperboloid sectors. The important feature of this concept is the proposed system of a few planes, dividing the roof structure into shell segments. This concept requires significant oblique cuts and a big transformation degree of all folded sheets.

**Figure 10:**
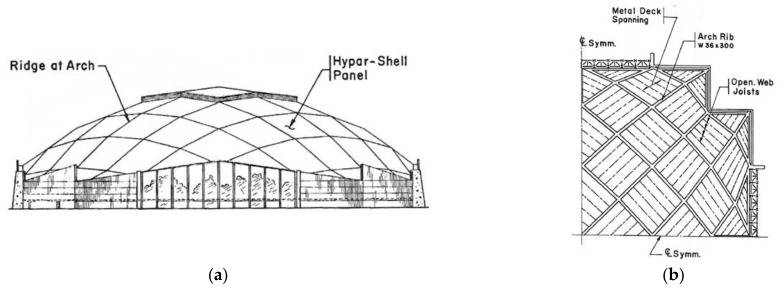
The second structure proposed by Biswas and Iffland [27]: (**a**) elevation; (**b**) framing plan of a quarter of the structure.

In the second concept, quadrilateral hyperbolic paraboloid sectors divided by the spherical system of planes are used (Figure 10a,b). The twist degree of all complete hyperbolic paraboloid sectors is rather small compared to the previous structure.

**Figure 11:**
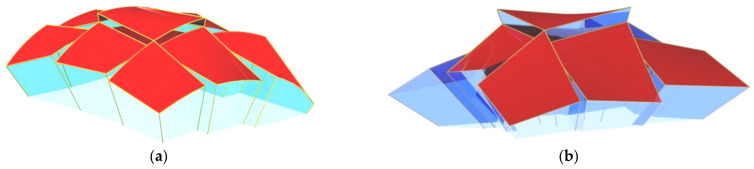
Complex building free-forms roofed with multi-segment shell structures and supported by (**a**) curved; (**b**) straight directrices.

Prokopska proposed various ways to increase the attractiveness of the ribbed shell structures. These are as follows: (1) areas of discontinuity between the adjacent steel segments, filled with, for example, glass panels (Figure 11); (2) green plant gardens placed on the transformed segments; and (3) communication routes between the segments [28]. To create attractive regular complex structures composed of many complete roof shells, he developed a geometric method [26] of creating a spatial polyhedral reference net arranged on various regular smooth surfaces of positive, negative or zero Gaussian curvature. The division of the network into spatial tetrahedral meshes allows one to easily locate single transformed folded shells and bar constructions supporting these segments in the three-dimensional space.

The above-mentioned problems show the wide possibilities of using the specific orthotropic geometric and mechanical properties of folded sheets in rational shaping of the transformed roof coatings [29,30]. A respective analysis should be carried out at different levels: single points, walls or folds, smooth shell models, and ribbed structures composed of many smooth transformed shell segments [31,32]. The presented issues indicate many unexplored areas in the field of effective elastic transformations of thin-walled folded roof shells and great possibilities of geometric and mechanical analysis at many different levels of accuracy.

Parameterization of the transformed shapes enables one to computationally search for attractive unconventional building free forms [22,26,33] and innovative structural systems intended for the investigated building free forms. Obrębski [34] developed a few methods for shaping very diversified shell rod structures. Rebielak [35] developed steel rod structural systems supporting flat roof covers composed of corrugated sheets. Abel and Mungan [36] present many examples of the construction systems associated with shaping many diversified roof shells and building free forms. Nominally plane folded steel sheets transformed plastically into shell shapes are used as curved supports for shell panels of entire roof covers [37]. In this way, convex roof shells characterized by a positive Gaussian curvature are created. Nominally plane folded steel sheets transformed elastically into shell shapes are used as suspended roof structures [38,39].

## 3. The Aim and the Scope of the Research

The main aim of the article is to present the geometric and mechanical properties of the nominally flat thin-walled folded sheets, which have a decisive influence on the way of shaping various unconventional corrugated transformed roof shells composed of these sheets. The basic geometrical quantities studied are the changes in width and twisting degree of a shell fold along its length. The essential mechanical quantities examined are stresses, strains and constitutive relationships between these stresses and strains.

A special role in creating geometric models of the transformed roof shells is played by the possibility of shaping such folded shells by means of regular smooth ruled surfaces to which orthotropic material properties of the corrugated sheets are assigned. Knowledge of the above-mentioned properties of the folded transformed sheeting allows one to derive displacement equations of smooth hyperbolic paraboloid sectors modeling the sheeting in various configurations. Then, it is going to be possible to develop a method of shaping the transformed roof sheeting based on the displacement equations. In line with the above purposes, a concept of presenting the vital geometric and mechanical properties as the basis for the development of such a method was created.

Similarly, the possibilities and limitations of the nominally flat thin-walled corrugated steel sheets adopted as a universal structural and filling material in the formation of roof coverings are presented. This material allows for the rational shaping of complete and complex sheeting characterized by unconventional effective ruled shell forms. The rationality and attractiveness of the transformed shell sheeting result from the specific orthotropic geometric and mechanical properties of the folded sheets used in designing and creating the shells. In order to obtain a single transformed roof shell, the folded sheets are joined into a nominally flat folded strip which is transformed into a ruled shell form so that its initial effort resulting from this transformation is minimized. The emergent initial stresses reach high values of about 100 MPa or even close to those referring to the yield point if they are not reduced by means of the appropriate calculation method, way of loading and technique of fixing the sheets to the roof construction resulting from the specific orthotropic properties of the folded sheets.

As a result, a freedom of the width and height increments of each transformed fold is assured to reduce these initial stresses and achieve the effective shape transformations. The next goal of the presented detailed analysis on the geometric and mechanical properties of the transformed sheets is to develop some rules for minimizing the initial stresses caused by the shape transformations. There are only considered the coatings, the transformations of which are the result of unfolding and supporting the transverse fold’s ends by two mutually skew straight directrices. Therefore, there are analyzed twist transformations whose twist degrees depend on the mutual inclination and distance of the roof directrices.

## 4. The Concept of the Research

The essential step of the research is the analysis of the orthotropic geometric and mechanical properties of the thin-walled folded sheets in order to select those properties that have a decisive influence on the form and work of the sheets in the transformed roof shells. The next step concerns the parameters describing these properties, so that they allow one to shape rational transformed sheet forms as (a) structural rod elements (for example, thin-walled beams), and (b) filling shell elements as, for example, an outside roof cover or a lost shell roof formwork. The presented analysis is based on the results of the laboratory tests related to the different transformation degrees and types of the profiles used for the transformed experimental shells.

The obtained results related to the geometric analysis of the shape properties of the transformed folded sheets treated as a two-dimensional orthotropic material are the basis for deriving the geometric stiffness matrix [40] and elasticity constants of various transformed shell sheeting. Thus, the shell shapes defined in a three-dimensional space by means of this material are described by two curvilinear coordinates.

From the above assumption, it follows that the transformed shell coverings are to be modeled by means of various smooth sectors and characteristic lines of regular warped surfaces [33]. The analyzed basic geometric elements are contracting lines, edge lines, supporting lines and central sectors of warped surfaces. The standardized variables are the lengths of the above-mentioned lines and the areas of warped surfaces modeling the entire transformed shells or their individual folds. In order to define the zones of compression and tensile stresses appearing in each shell fold of a transformed roof, the width changes along the length of the fold are determined by means of the above variables.

The level of the accuracy of the analysis related to the geometric properties of the entire transformed folded shells or their complete folds does not have to be great. It may be limited to central sectors and lines distinguished on two-dimensional warped surfaces [19]. On the other hand, the level of the analysis related to a stress state of each shell fold must refer to the selected points characterizing all individual walls of the fold and their common edges [41]. Therefore, the level of the detailed mechanical analysis associated with the transformed shells is significantly greater than the previous one.

In order to elaborate a quantitative description of the effective shape transformations of the corrugated shells, such parameters related to the mechanical properties of the folded sheets as shear or normal stresses and strains are examined [42,43]. The effect of these transformations is to be such a state of equilibrium in which the effort of each shell fold is going to be the smallest possible and the work of the internal forces is balanced out [44]. Therefore, the following are considered: (a) the type of the stress state, e.g., whether it is pure shear or complex stress state [45], (b) loading conditions, and (c) supporting conditions adopted for the entire cover and its complete folds. As a result, the interdependences between the parameters describing the form and the stress state of each folded shell, the transformation degree and the adopted supporting conditions of the shell are sought.

The invented dependencies should describe the subsequent momentary states of equilibrium of all shell folds, obtained as the result of the effective subsequent transformations corresponding to the increase in the transformation degree. A quantitative description of these configurations should allow one to derive displacement equations of each transformed shell using smooth sectors of regular warped surfaces. On the basis of the above equations, it becomes simple to calculate the strains, stresses and the form of the designed transformed shell, including the length of its directrices, edge lines and line of striction.

The big variety of the employed sheet types, ways of joining these sheets in the roof shells, methods of modeling, loading and supporting of the designed shells induces the development of a universal accurate thin-walled FEM computer model [41]. Such a model was developed and then initially configured based on the results of the experimental research [23]. This model is used in the presented research. It is planned to be used in further studies, too.

## 5. Geometric Characteristics of the Transformed Folded Roof Shells

**Figure 12:**
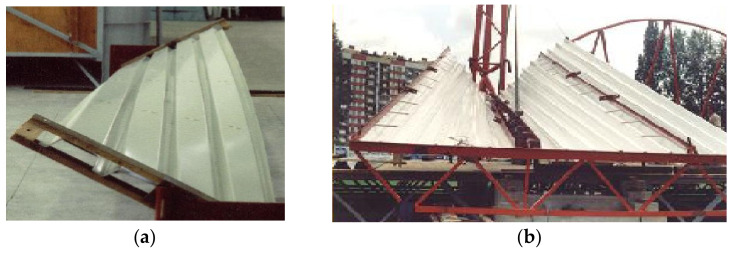
Transformed folded sheets characterized by: (**a**) free arched longitudinal edges; (**b**) stiffened straight edges.

The nominally flat folded sheets are subject to the initial transformations to achieve their shell shapes. The unstiffened longitudinal edges of the transformed sheets become convex arcs (Figure 12a). If the longitudinal edges are stiffened with special rigid profiles or other profiled sheets, Figure 12b, then the edges and longitudinal axes of the subsequent folds can be regarded as straight. It is necessary to provide all transformed shell folds with appropriate boundary conditions. In particular, the technique of unfolding the sheets onto the roof directrices, joining the sheets longitudinally and fixing the folds to the roof directrices should assume the pre-stresses and pre-strains of each shell fold to be the smallest possible regardless of the transformation degree. In addition, the longitudinal edges of all shell folds ought to stay straight.

Uncontrolled shape transformations of the sheets lead to large deformations of their walls: flanges and webs resulting from the small transverse bending stiffness and the twist stiffness around the longitudinal axis of each fold. On the other hand, the relatively high deformations of the walls and high transformation degree of each shell fold are necessary to obtain deep, medium and even some shallow shells. They cause non-linear relationships between different dimensions characterizing the shell form of each transformed folded sheeting. It is advisable to reduce the level of the initial effort of the fold by limiting the value of its twist degree or using a method assuring the freedom of the transversal width increments of each shell fold. In this case, the shape transformation is called effective.

**Figure 13:**
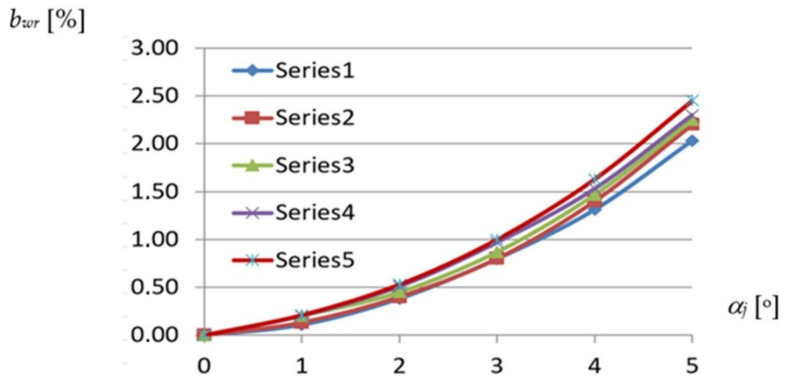
Nonlinear relations between *b_wr_* relative width increments of fold’s crosswise ends and *α_j_* unit angle obtained for various profiles of a shell fold supported by straight directrices: Series1—TR50 × 0.75 mm × 8.0 m; Series2—T55 × 0.88 mm × 6.2 m; Series3—TR85 × 0.75 mm × 5.0 m; Series4—TR136 × 0.88 mm × 6.0 m; Series5—T160 × 0.75 mm × 6.6 m.

The most important dependence used in the description of the geometrical properties of each effectively transformed shell fold is defined between the width of the fold along the roof directrix and its transformation degree (Figure 13) adopted as the unit twist angle of the fold resulting from the inclination of two skew directrices supporting the fold at its crosswise ends (Figure 12). The denotation TR 50 mm × 0.75 mm × 8.0 m means that the folds of the examined sheets are trapezial—TR and the height, thickness and length of these sheets are respectively equal to 50 mm, 0.75 mm and 8.0 m.

Total twist angle *α_c_* of a shell fold or sheet, shown in Figure 5 as ϕ, is defined as the angle of twist of the opposite transverse fold or sheet’s ends passing compatibility with the directrices of a shell. The unit twist angle *α_j_* is defined as the total twist angle *α_c_* divided by the length of the fold or sheet measured in meters. On the basis of the observations made, the unit twist angle defining the transformation degree is assumed to be constant along the fold’s length. To precisely study the obtained relationships and make the analysis independent of the number and width of the folds in the considered sheets, the relative width increments are considered. The relative *b_wr_* width increments of any shell fold are defined as the quotient of its absolute *b_w_* width after transformation to the width *b*_0_ of the fold before the transformation as follows:

*b*_*wr*_ = 100 × *b_w_*/*b*_0_ (1)

Each of the lines, Series1 to Series5, introduces a relationship obtained for the reference fold of the experimental folded shell of a strictly defined profile. For example, the Series1 line represents the relationship between *b_wr_* and *α_j_* of the reference fold defined for the TR 50 m × 0.75 mm × 8.0 m profile. This line was determined on the basis of six points whose coordinates are the values of the unit twist angles and the values of the relative width increments of the above-mentioned fold. These values were obtained for the successive configurations of each experimental shell generated by means of an increase in the transformation degree of this shell. Therefore, for five different shells being characterized by a different profile, five reference folds were determined. The presented characteristics of these folds were examined in six subsequent configurations of each respective transformed experimental shell, where each configuration was generated by increasing the transformation degree of the shell. Each quantity describing the respective property of the reference fold was calculated on the basis of the corresponding quantities measured during the tests for several adjacent folds of the examined experimental shell.

The above diagram, shown in Figure 13, shows a relatively small variation in the influence of the profile type on the fold’s shell form in the range of the unit twist angle of up to 3.5°. In this case, the variation causes an insignificant differentiation of the fold’s width increments comparable with the measurement accuracy of about ± 0.5 mm, which corresponds to the value of *b_wr_* = ± 0.2%.

**Figure 14:**
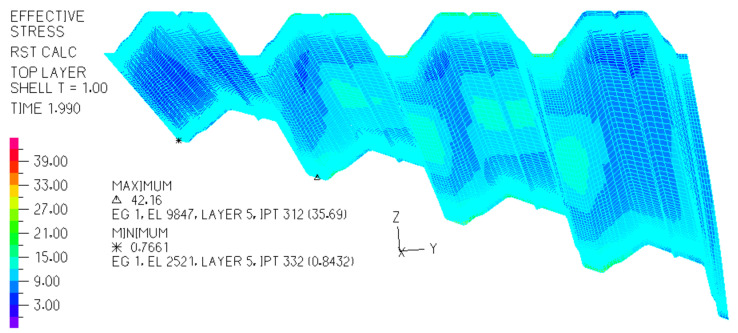
A computational thin-walled mechanical model of a nominally plane folded sheet transformed initially into a shell shape and the graphical expression of the “effective” stresses in MPa on its top surface.

**Figure 15:**
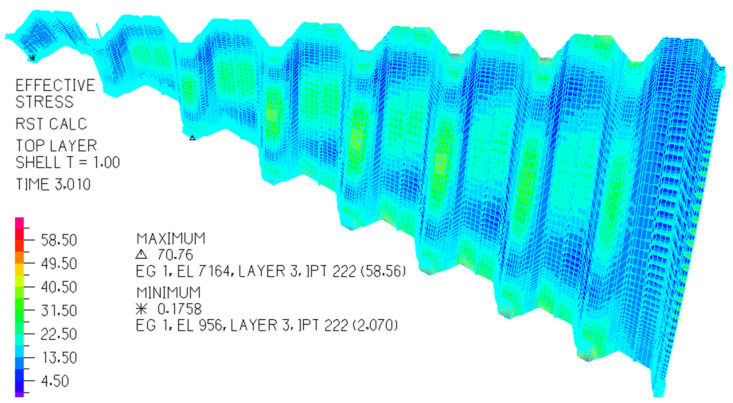
A computational thin-walled mechanical model of nominally plane folded sheeting transformed initially into a shell shape, and the graphical expression of the “effective” stresses in MPa on its top surface.

The above dependencies were obtained by means of the experimental tests on the experimental stand [14,20,42] (see Figure 6). They made it possible to configurate the elaborated thin-walled FEM computer models [23] (Figure 14 and Figure 15) used in computer simulations whose results are presented in the next section.

On the basis of the analysis made to date, it should be stated that the folded sheets with (a) free longitudinal edges (Figure 12a), and (b) stiffened longitudinal edges (Figure 12b) are characterized by mutually different geometrical relationships [14]. Both types of the sheets can be modeled with the same type of ruled surface, but the sectors of this surface corresponding to the above sheets must be different [20]. In the first case, the free longitudinal arcuate edges are modeled with curved lines. In the second case, the longitudinal edges are straight. The above two types of folded sheets cause different lengths of their contracting lines, and different lengths of their supporting lines passing along their crosswise roof edges. Therefore, the sheets with free longitudinal edges should be converted to sheets with straight longitudinal edges by means of the same type of smooth surface.

**Figure 16:**
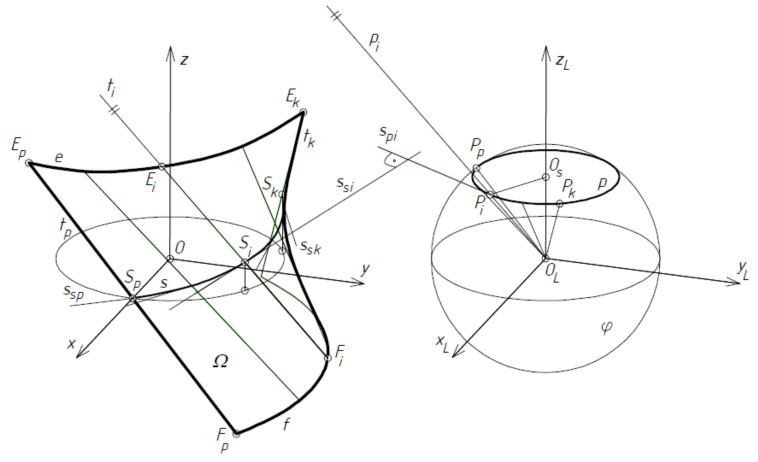
Central sector *Ω* determined on the basis of line of striction *s* and spherical indicatrix *p* determining warped surface.

In a general case, the equation of ruled surface *Ω* modeling one fold, sheet or whole transformed shell is as follows:

*r*(*u*,*v*) = *e*(*u*) + *p*(*u*) × *v* (2)

where *r*(*u*,*v*) = [*x*(*u*,*v*), *y*(*u*,*v*), *z*(*u*,*v*)] is the vector of the position of any point on a ruled surface *Ω*, *e*(*u*) is the vector of the position of any point on directrix *e* (Figure 16), and *p*(*u*) is the unit director vector of ruling *t_i_*. All vectors *p*(*u*) can have one common origin at point *O_L_* and determine that spherical indicatrix *p(u)* contained in sphere *φ* of the unit radius and center *O_L_*, and *u*, *v* are curvilinear coordinates of *Ω*.

The location of any point on the line of striction *s*(*u*) of *Ω* in relation to directrix *e*(*u*) of *Ω*, can be determined by the following formula:

(3) $$v\left(u\right)=\frac{e^{\prime }\left(u\right){\;\times \;p}^{\prime }\left(u\right)}{{\left(p^{\prime }\left(u\right)\right)}^{2}}$$

where *s*(*u*) is the line of striction composed of the central points of all rulings *t_i_* of *Ω*, and *v* is the parameter describing the position of any point of *s(u)* on the respective ruling *t_i_* in relation to the adopted directrix *e*(*u*). If *s(u*) is adopted as directrix *e*(*u*), the following condition must be satisfied:

(4) $$v\left(u\right)=\frac{s^{\prime }\left(u\right)\times p^{\prime }\left(u\right)}{{\left(p^{\prime }\left(u\right)\right)}^{2}}=0$$

which results from comparing Equation (3) to zero. From Equation (4), it follows that the *s′*(*u*) straight line tangent to *s*(*u*) and the straight line *p′*(*u*) tangent to the spherical indicatrix *p*(*u*) of *Ω* have to be perpendicular to each other, so

(5) $$s^{\prime }\left(u\right)\times p^{\prime }\left(u\right)=0$$

If Equation (5) is preserved, the function *e*(*u*) in Equation (2) should be replaced by $s\left(u\right)$ representing a line of striction modeling the contraction of the designed folded shell.

**Figure 17:**
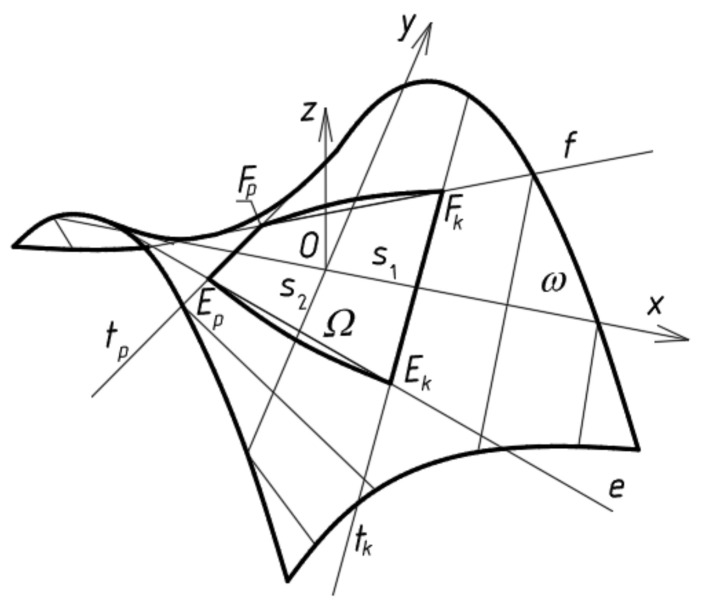
Z-axis-symmetrical sector *Ω* of right hyperbolic paraboloid *ω* adopted as a model for a corrugated transformed shell.

A special type of ruled surfaces is used in the further considerations. This is right hyperbolic paraboloid, Figure 17, whose mathematical equation is as follows:

(6) $$\frac{f}{a\times b}x\times y=z$$

where *f*, *a* and *b* are the constants determining the right hyperbolic paraboloid *ω*, its sector *Ω* and line of striction *s*_1_.

**Figure 18:**
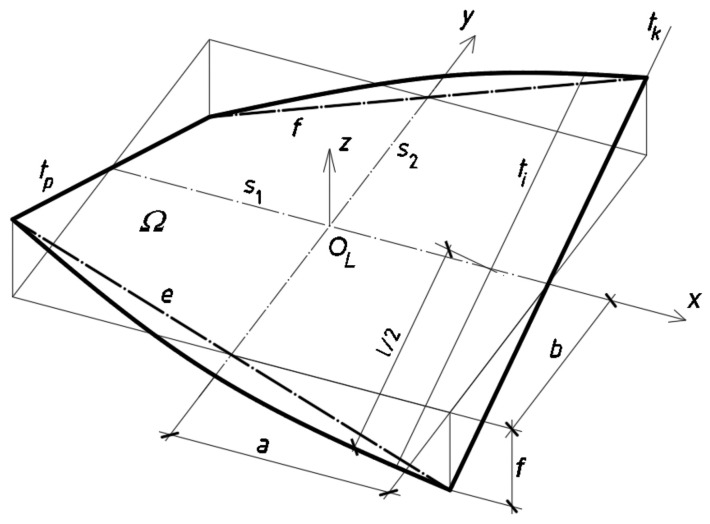
Geometrical characteristics of a *z*-axis-symmetrical sector *Ω* of a right hyperbolic paraboloid *ω* adopted for the smooth model for a transformed folded shell.

A parametric equation of right hyperbolic paraboloid can be created by means of Equation (2) as follows:

(7) $$x\left(u,v\right)=u\;y(u,v)=v\times \frac{b}{\sqrt{b^{2}+{\left(\frac{f}{a}\right)}^{2}\cdot u^{2}}}\;z(u,v)=v\times \frac{\frac{f}{a}\cdot u}{\sqrt{b^{2}+{\left(\frac{f}{a}\right)}^{2}\cdot u^{2}}}$$

where *a*, *b*, and *f* are the constants characterizing the right hyperbolic paraboloid sector *Ω*, Figure 18. The following parametric equations of its line of striction *s*_1_ are used in the modeling shell folds

(8) $$x\left(u\right)=u\;y=0\;z=0$$

The second line of striction of the paraboloid is a straight line *s*_2_ perpendicular to *s*_1_ and contained in the plane (*x*,*y*). The line *s*_2_ is not employed in the further analysis. The above parametric equations are convenient for defining some quantitative geometrical relationships describing the basic properties of right hyperbolic paraboloids because, for example, the parameter *v* expresses the position of a point along the length of each ruling, so it changes from −*l*/2 to *l*/2 for all subsequent folds of each transformed shell.

**Figure 19:**
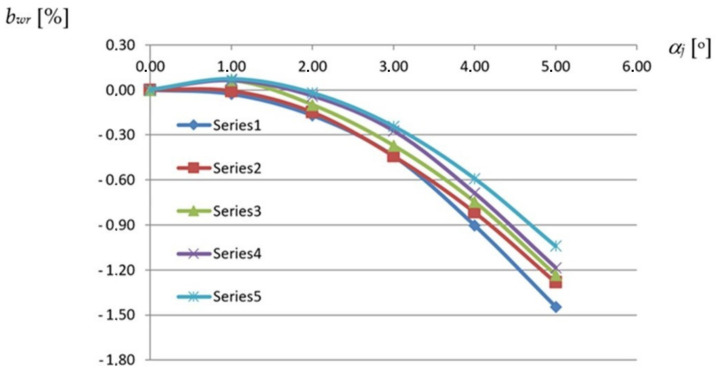
Nonlinear relations between the relative width increments *b_wr_* at a half along the fold’s length and the unit angle *α_j_* obtained for various profiles of a shell fold supported with straight directrices: Series1—TR50 × 0.75 mm × 8.0 m; Series2—T55 × 0.88 mm × 6.2 m; Series3—TR85 × 0.75 mm × 5.0 m; Series4—TR136 × 0.88 mm × 6.0 m; Serise5—T160 × 0.75 mm × 6.6 m.

On the basis of the relative width increments *b_wr_* presented in Figure 13 and referring to the transverse edges of the transformed folds of different profiles, the minimum widths *b_wr_* of these folds at a half along their length were calculated assuming that each fold is modeled by a central sector of a right hyperbolic paraboloid given by Equation (7) and its line of striction by Equation (8). The diagram (see Figure 19) presents relations between the relative width increments *b_wr_* measured at a half along the fold’s length and the unit twist angle of the reference shell folds assigned to various types of profiles. The relative width increments *b_wr_* of these folds were calculated using Equation (1).

The relative *p_wr_* neutral surface increments of any shell fold are defined as the quotient of its absolute *p_w_* neutral surface area after the transformation to the plane neutral surface area *b*_0_ of the fold before the transformation as follows:

*p_wr_* = 100 × *p_w_*/*p*_0_ (9)

The Series1 line corresponds to the same TR50 × 0.75 mm × 8.0 m reference fold for which the same-named line was created in Figure 13. The difference between these lines is that the values shown in Figure 13 are the width increments measured along the transverse ends of the reference fold while the values presented in Figure 19 are the width increments measured at a half along the length of this fold. The above increases are one of the basic variables on which the designed displacement method of shaping the considered shells is planned to be based.

Since all effectively transformed folds contract in a half along the fold’s length in a way dependent on the type of the fold’s profile, the achieved relations assist in determining the smooth shell models of each shell fold by means of the line of striction, especially its shape and length. The absolute values of these increments are approximately halves of the positive width increment values at the stretched crosswise ends of the shell folds. Although the relations obtained between these increments and the degree of twist of the folds are non-linear, some disturbances in the course of the complete curves corresponding to the folds of the higher profiles and the smaller transformation degrees can be noticed. Such anomalies appear due to the significant influence of the weight on the width increments of each fold at a half along its length.

The areas of central sectors *Ω* of right hyperbolic paraboloids defined by means of the above-mentioned relationships play a significant role in further geometric and strength analyses. The area surface of the sectors can be calculated as follows:

(10) $$P\left(u,v\right)=2\times \int_{0}^{ukp}\int_{-l/2}^{l/2}\sqrt{AA^{2}+BB^{2}+CC^{2}}dvdu$$

where the following Jacobi functions must be calculated:

(11) $$AA=\left[\begin{array}{cc} \frac{\partial y(u,v)}{\partial u} & \frac{\partial z(u,v)}{\partial u}\\ \frac{\partial y(u,v)}{\partial v} & \frac{\partial z(u,v)}{\partial v} \end{array}\right],\;BB=\left[\begin{array}{cc} \frac{\partial z\left(u,v\right)}{\partial u} & \frac{\partial x\left(u,v\right)}{\partial u}\\ \frac{\partial z\left(u,v\right)}{\partial v} & \frac{\partial x\left(u,v\right)}{\partial v} \end{array}\right],\;CC=\left[\begin{array}{cc} \frac{\partial x(u,v)}{\partial u} & \frac{\partial y(u,v)}{\partial u}\\ \frac{\partial x(u,v)}{\partial v} & \frac{\partial y(u,v)}{\partial v} \end{array}\right]$$

where *l* is the length of each fold, and *ukp* is the function describing the position of the edge ruling *t_k_* of *Ω* defined on the hyperbolic paraboloid. Thus, the function describes the width of the entire transformed shell sheeting.

Centers of gravity of some selected cross-sections of each shell fold are calculated to determine the longitudinal axis of the fold. The longitudinal axes of all folds define a smooth neutral surface of the entire shell and each complete fold. The neutral surface can be taken as a simplified smooth model of the entire transformed shell and treated as a sum of the complete fold’s models. The surface area of this model can be calculated using Equations (10) and (11). This model of the transformed shell can be used together with another smooth model that is created on the basis of the longitudinal straight edges of all subsequent shell folds, see Figure 5. A rectangle is a simplified smooth model of the flat fold before its transformation. The area of the rectangle is denoted as *p_0_*. The neutral surface area of the fold after its transformation is denoted as *p_w_*. The relative neutral surface area increments *p_wr_* of the fold can be calculated using Equation (9).

**Figure 20:**
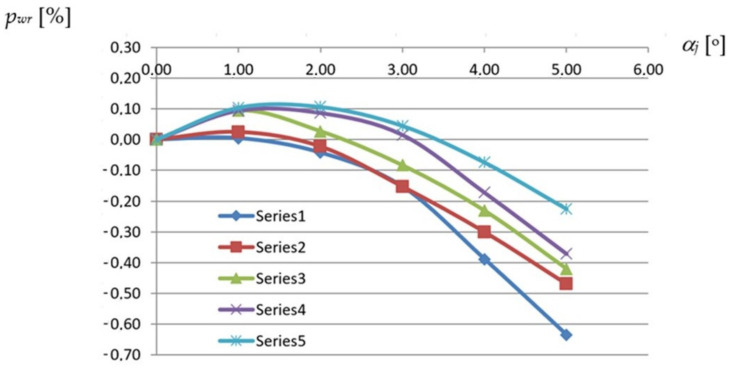
Nonlinear relations between the relative *p_wr_* increments of the surface area and the unit twist angle *α_j_* of a shell fold supported by two straight directrices obtained for various profiles: Series1—TR50 × 0.75 mm × 8.0 m; Series2—T55 × 0.88 mm × 6.2 m; Series3—TR85 × 0.75 mm × 5,0 m; Series4—TR136 × 0.88 mm × 6.0 m; Series5—T160 × 0.75 mm × 6.6 m.

The graph presented in Figure 20 shows that the *p_wr_* relative increments of the neutral surface area of each transformed shell fold nonlinearly decrease with the increase in the fold’s transformation degree. The obtained results depend on the size of the fold. The *p_wr_* relative increment of the neutral surface of each shell fold is defined as the quotient of the *p_w_* absolute neutral surface increment of the fold to its area *p_0_* before the transformation.

Each of the curves shown in Figure 20 corresponds to a reference fold designated for a different type of profile. For example, the Series1 line corresponds to the same reference fold to which the Series1 lines, shown in Figure 13 and Figure 19, were previously assigned. Each curve from Figure 20 was derived on the basis of five points whose coordinates are different values of the unit twist angle and the relative increase in the neutral surface area of the fold. These points correspond to the selected subsequent configurations of the appropriate experimental transformed shell characterized by a strictly defined profile. These configurations were generated by gradually increasing the twist degree of the experimental shell.

Some disturbances in the course of the above curves corresponding to the folds of the higher profiles can be noticed for smaller twist degrees. This fact results from the influence of the weight of the transformed folds on the size of the surface areas and that the twist of each hyperbolic paraboloid fold nonlinearly varies along its length. The variability can accurately be expressed by the dependence of the *α_i_* rotation angle of the successive cross-sections along the fold’s length according to the following formula:

(12) $$\alpha_{i}=\mathrm{atan}(\frac{f}{a\cdot b}\times y)$$

calculated on the basis of the geometrical properties of central sector *Ω* of right ruled paraboloid, where *f*, *a* and *b* are the constants of *Ω* used in (6).

An additional increase in the width of the fold’s transverse end induced by, for example, adjusting this end to the length of the directrix may cause an important change in the position of the contraction line on *Ω*. The inclination of the contraction line to the ruling *t_i_* may also change. As a result, the ruled surface is no longer a right hyperbolic paraboloid. In this way, we obtain the possibility of increasing the variety of the unconventional transformed folded shells forms. The models of such transformed shell sheeting can be adopted in the form of warped surfaces of various types, including conoids or cylindroids. The models can be defined using Equation (2) and their rulings can be determined by means of Equation (12).

The neutral surface areas of the transformed folds and their compressed and stretched sectors are important when calculating the work of the internal forces occurring in each folded shell. The compressed area is related to the line of contraction. The tensile areas refer to the supporting directrices.

The location of the contraction of a transformed shell at the length of all transformed folds must be determined by means of a proper calculation method based on the specific orthotropic properties of the corrugated shells. In addition, respective supporting conditions and ways of loading must be adopted. Each shell fold is in an optimal state of equilibrium if it can freely deform in its transverse directions and its contraction appears halfway along the fold’s length. In the case of straight directrices, the initial stress level is reduced to the smallest possible and the considered central sectors of hyperbolic paraboloids are characterized by straight lines of striction perpendicular to all shell folds.

## 6. Mechanical Properties of the Transformed Folded Roof Shells

Since the transformed folds are characterized by small transverse bending stiffness and small torsional stiffness around the longitudinal axis, nonlinear geometric relationships are observed. A few basic geometric relationships were presented in the previous section. In the present section, some nonlinear interdependences occurring between the mechanical properties and the unit twist angle of the twisted folded sheets are presented.

**Figure 21:**
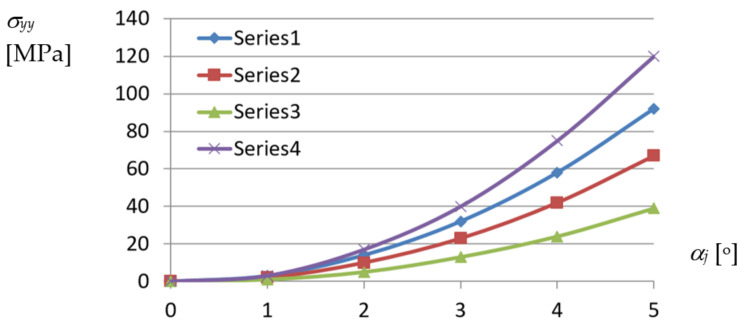
Relations between *σ_yy_* normal stresses acting orthogonally to longitudinal axes and *α_j_* unit angle of the transformed sheets of the following profiles: Series1—TR50 × 0.75 mm × 6.2 m; Series2—TR60 m × 0.75 mm × 6.2 m; Series3—TR130 × 0.75 mm × 6.2 m; Series4—TR85 × 0.75 mm × 5.0 m.

Based on the experimental studies carried out in the Rzeszow University of Technology hall [19,42], there was elaborated a diagram composed of curves corresponding to various profiles, representing relations between the normal stresses acting orthogonally to the directions of the longitudinal axes and *α_j_* unit twist angle of a shell fold (Figure 21).

**Figure 22:**
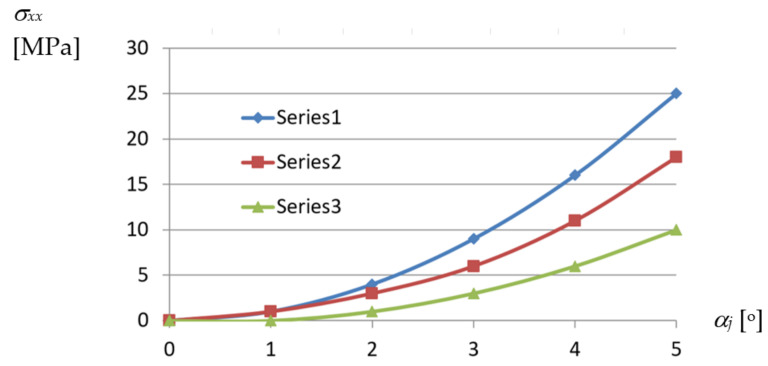
Relations between *σ_xx_* normal stresses acting along longitudinal axes and *α_j_* unit angle of numerically transformed sheets of the following profiles: Series1—TR50 × 0.75 mm × 6.2 m; Series2—TR60 × 0.75 mm × 6.2 m; Series3—TR130 × 0.75 mm × 6.2 m.

The curves presented in Figure 22 and corresponding to various profiles express the relations between the *σ_xx_* normal stresses acting along the longitudinal axes of the shell folds and *α_j_* unit angle of these folds.

**Figure 23:**
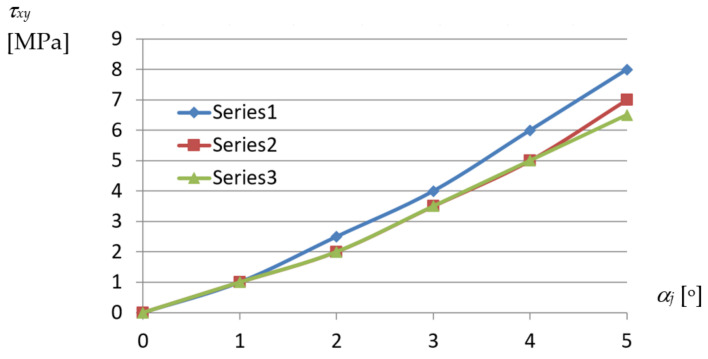
Relations between *τ_xy_* shear stresses acting in fold’s walls and *α_j_* unit angle of numerically transformed sheets of the following profiles: Series1—TR50 × 0.75 mm × 6.2 m; Series2—TR60 × 0.75 mm × 6.2 m; Series3—TR130 × 0.75 mm × 6.2 m.

Similarly, the curves presented in Figure 23 represent the relations achieved between the shear stresses *τ_xy_* and the *α_j_* unit twist angle of the examined fold.

From the above diagrams, the following conclusion can be drawn. The unit twist angle of 3° induces important initial normal stresses of about 10 to 40 MPa in the directions passing transversally toward the fold’s longitudinal axes. Thus, the stresses have to be taken into account during the design process, for the unit angle higher than or equal to 3°. The observed shear *τ_xy_* and normal *σ_xx_* stresses, the latter acting along the fold’s longitudinal axes, are of little importance compared to *σ_yy_*. The big differences in stresses obtained for different profiles, referring to the same measure of the fold’s unit twist angle, are noticed. The stresses non-linearly decrease with the increase in the profiles’ height.

**Figure 24:**
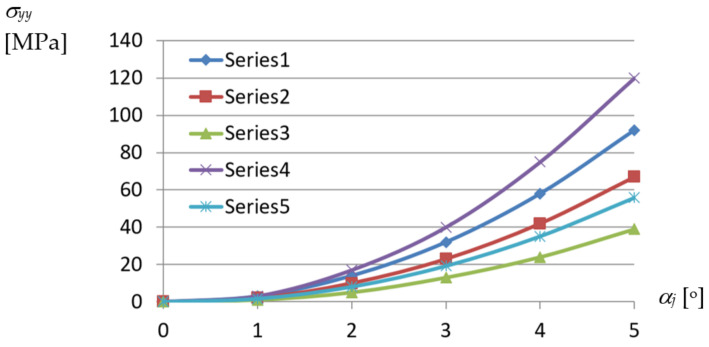
Comparison between the dependencies obtained for *σ_yy_* normal stresses acting orthogonally to fold’s longitudinal axes in relation to *α_j_* unit twist angle of the transformed sheets of profiles: Series1—TR50 × 0.75 mm × 6.2 m (called Serie1); Series2—TR60 × 0.75 mm × 6.2 m; Series3—TR130 × 0.75 mm × 6.2 m; Series4—TR85 × 0.75 mm × 5.0 m; Series5—TR85 × 0.75 mm × 5.0 m (the computational thin-walled model).

On the basis of the determined dependencies, an elaborated exact thin-walled mechanical model of a transformed fold of the examined TR 85 m × 0.75 mm × 5.0 m profile was configured. The model is used to describe the changes in the geometric and mechanical properties of various effectively transformed shell folds. A relationship obtained between the normal stresses acting transversely to the direction of an effectively twisted fold of the TR 85 m × 0.75 mm × 5.0 m type and the unit twist angle of the fold was achieved by means of this model (Figure 24). The relationship is represented by the curve labeled Series5. Other curves shown in this figure enable one to compare the achieved results of the experimental tests and computer simulations. All examined shell folds are fastened to the directrices with their lower flanges, only.

The highest normal stresses *σ_yy_* are assigned to the crosswise fold’s ends and directions passing transversally to the longitudinal axes of the shell folds. The nonlinear tendencies related to the increase in the unit twist angle and height of the examined folds are obvious. However, in the case of Series4 and Series5 referring to the same type of profiles employed, the results are too divergent, most likely because of the actual supporting conditions obtained at the shell fold’s ends fastened to the directrices. In the case represented by the Series4 line, the way of fixing the fold’s end was too stiff, so the freedom of the fold’s width increments was not ensured. In contrast, the relation represented by the Serie5 curve is convergent with the other ones.

**Figure 25:**
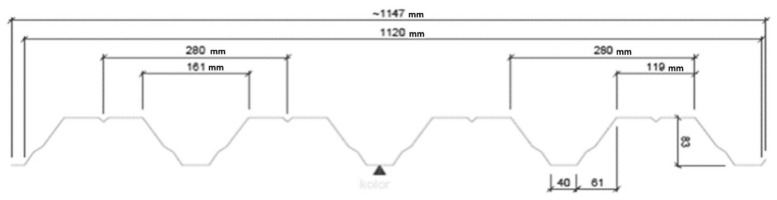
Geometrical features of the T85 × 0.75 profile used in the tests and computer simulations.

Since the results obtained by means of the thin-walled computer model were integrated and standardized by the results of the experimental tests, the mechanical properties of the variously transformed subsequent shell folds can be analyzed using this model to define an orthotropic material employed for shaping unconventional folded roof shell forms. It was assumed that the examined steel sheets modeled by means of the thin-walled computer model are of the T85 × 0.75 profile, see Figure 25, and 5.00 m length. The modulus of elasticity E = 2.05 GPa.

Despite the highly non-linear relationships presented in the above diagrams, the constitutive relations

**Figure 26:**
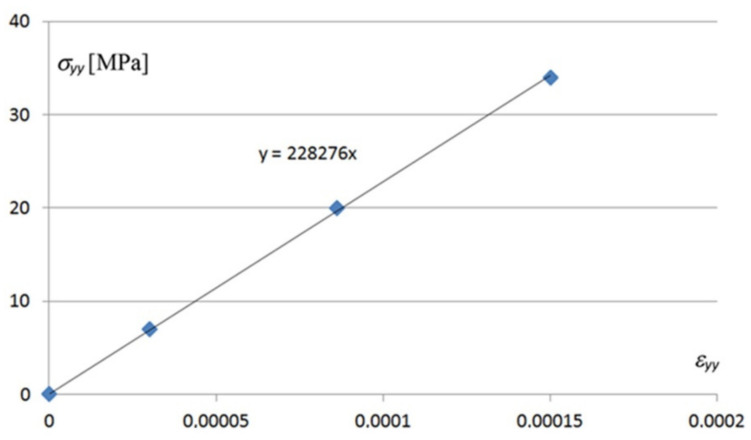
A linear relation between the *σ_yy_* normal stresses and the *ε_yy_* corresponding strains appearing at a point located at the crosswise end of a shell fold.

{*σ*} = [*E*] {*ε*} + {*σ*_0_} (13)

between stresses {*σ*} and corresponding strains {*ε*} appearing in the examined profile TR 85 are linear. Variable [*E*] is the stiffness matrix of the sheets and {*σ*_0_} is the vector of the initial stresses resulting, for example, from the folding of a smooth thin-walled sheet into corrugated plate. The main linear relation between normal stresses *σ_yy_* and corresponding strains *ε_yy_* appearing in the areas of the biggest values of these stresses is shown in Figure 26.

The methods based on small strains and big deformations, referring to cross-sections of the 4th code class [46], can be used for strength shaping of the transformed shells in the tested range of the shape twist transformation degree of up to 5°. Other linear constitutive relations, including the ones for the *σ_xx_* normal stresses and *τ_xy_* shear stresses are presented in the figures included in Appendix A.

**Figure 27:**
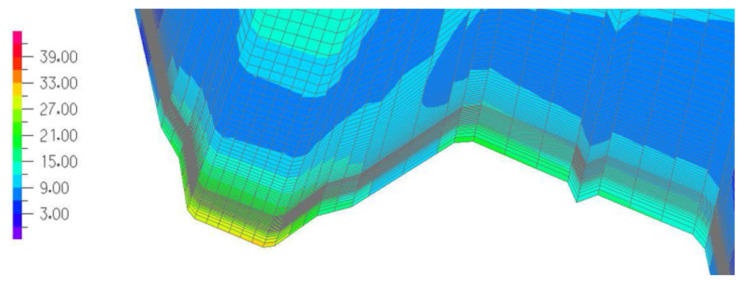
The map of the *σ_yy_* normal stresses acting orthogonally to the fold’s longitudinal axis, on the selected crosswise end of a shell fold.

A detail of the graphical representation of the normal stresses *σ_yy_* acting in the upper surface of the transformed accurate computational model of the TR 85 × 0.75 shell fold at its crosswise end is shown in Figure 27 in the form of a map. We can see how quickly the values of the *σ_yy_* normal stresses increase when we approach the fold’s crosswise end.

**Figure A1:**
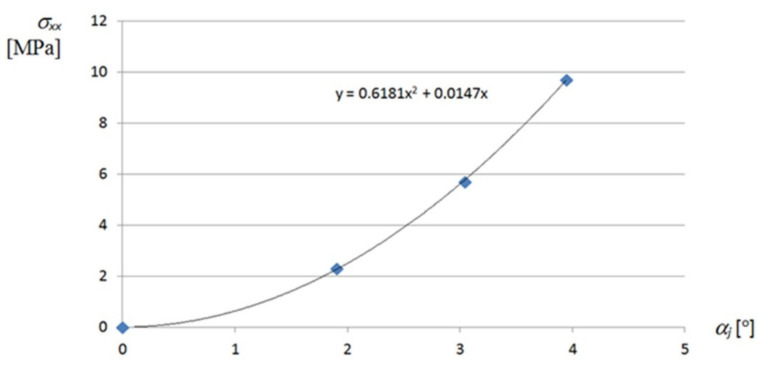
A non-linear relation between the *σ_xx_* normal stresses acting at the crosswise ends of an effectively twisted fold along the fold’s longitudinal axes and its *α_j_* unit angle.

A non-linear dependence of the normal stresses *σ_xx_* appearing at the transverse ends of the examined effectively twisted fold and acting along the direction of the fold on its unit twist angle *α_j_* is shown in Figure A1 included in Appendix A. From this diagram, it follows that the stresses reach much smaller values than *σ_yy_*.

**Figure A2:**
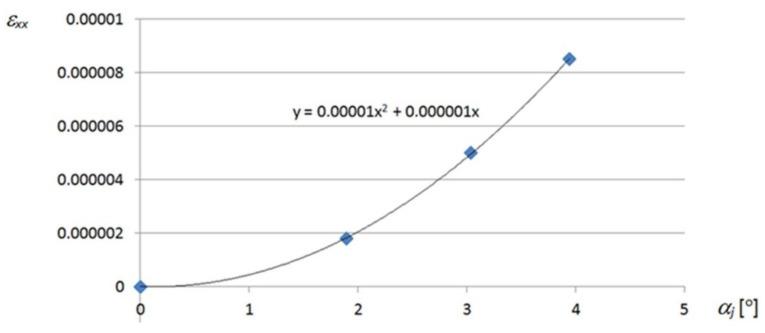
A non-linear relation between the strains *ε_xx_* corresponding to the normal stresses *σ_xx_* acting the along longitudinal axes and *α_j_* unit angle at the crosswise ends of an effectively twisted fold.

**Figure A3:**
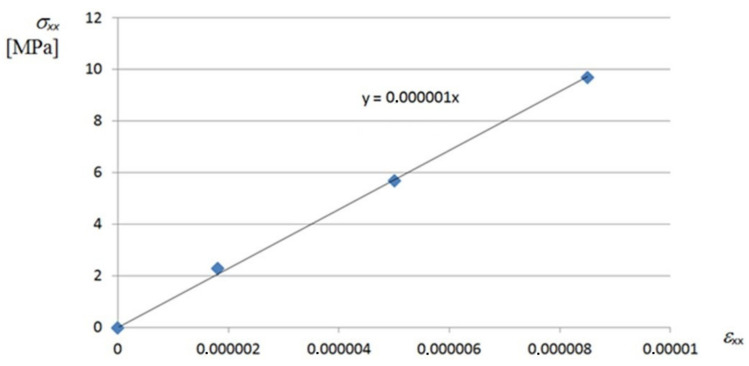
A linear relation between the *σ_xx_* normal stresses and corresponding *ε_xx_* strains obtained for elements located at the crosswise ends of a shell fold.

An analogous non-linear dependence of the strains *ε_xx_* corresponding to *σ_xx_* on the unit twist angle *α_j_* of the effectively transformed fold is shown in Figure A2. In turn, a linear relation between the *σ_xx_* normal stresses and corresponding *ε_xx_* strains, for the most deformed walls located at the crosswise ends of the shell fold, can be observed in Figure A3.

**Figure 28:**
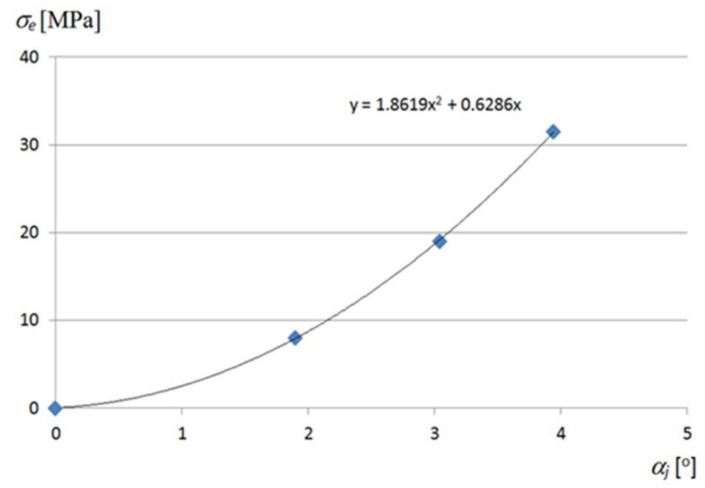
A nonlinear relation between the “effective” *σ_e_* stresses and *α_j_* unit angle at the crosswise ends of an effectively twisted fold.

**Figure A4:**
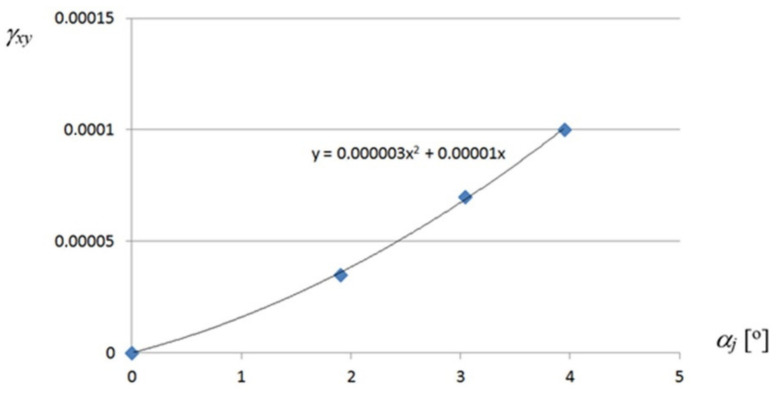
A nonlinear relation between the strains *γ_xy_* and *α_j_* unit angle at the crosswise ends of an effectively twisted fold.

**Figure A5:**
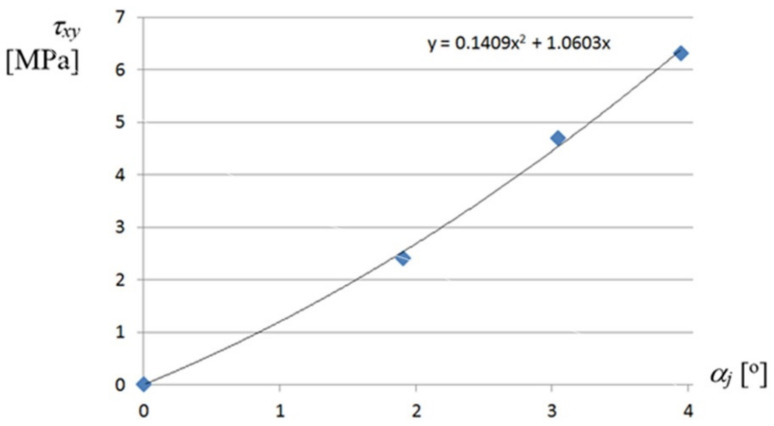
A non-linear relation between the shear stresses *τ_xy_* and *α_j_* unit angle at the crosswise ends of an effectively twisted fold.

**Figure A6:**
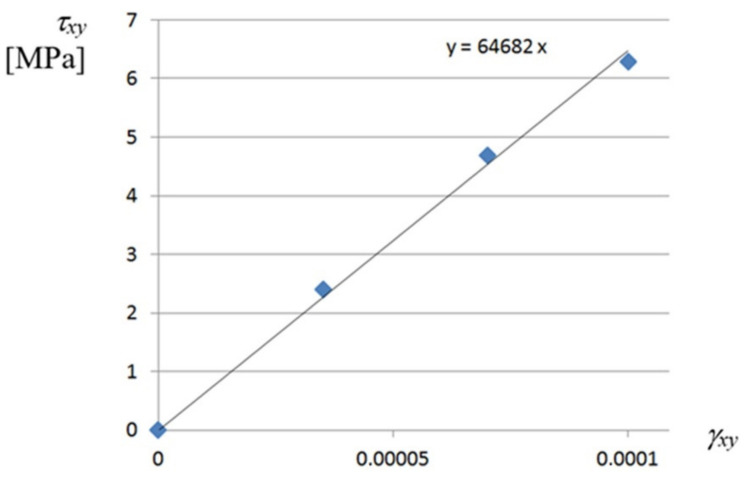
A linear relation between the *τ_xy_* shear stresses and *γ_xy_* corresponding strains for elements located at the crosswise ends of a shell fold.

The shear stresses *τ_xy_* and corresponding strains *γ_xy_* of the most deformed fold’s walls also take small values, Figure A4 and Figure A5. They reach the maximum values of 6.0 MPa and 0.000072 for *α_j_* = 3.94°, which allows one to assume that these values are negligible for the calculation of the stress–strain state of the transformed folds. The relationship obtained between *τ_xy_* and *γ_xy_* is linear, too (Figure A6). A nonlinear relation between the “effective” Huber–Mises–Hencky stresses *σ_e_* and the unit twist angle *α_j_* achieved for the walls located at the crosswise fold’s ends is shown in Figure 28.

On the basis of all aforementioned diagrams, it can be concluded that the twist of each shell fold by the unit angle of 3° to 5° induces its significant effort, affecting the mechanical properties of the fold. Such values of the transformation degree allow quite significant differences in the shapes, curvatures and contractions of the designed variously transformed shell roofs. Similarly, the initial strains of such deformed folds are relatively small compared with these referring to the yield point or even the elastic limit of steel. The presented examples allow the designer to use linear constitutive relationships in the analyzed ranges of the transformation degree.

The conducted analysis shows that it is advisable to search for such effective transformations that the level of the normal stresses *σ_yy_* would be reduced to the minimum. For this purpose, it is necessary to assure the freedom of the fold’s width increments determining the linear constitutive relationships between the stresses and strains. The respective calculation methods, loading conditions and supporting conditions are needed.

From the relationships presented in this section, some additional, important conclusions can be drawn. The complex states of stresses archived for the examined twisted folded shell sheeting supported by straight directrices cannot be reduced to pure shear. The *σ_yy_* normal stresses acting perpendicularly to the old’s directions play the most important role in this state. The state causes a precisely defined position and length of the contraction line on the transformed shell. The aim is to calculate the length and such a position of the contraction that the contraction passes through the middle of each shell fold. In this case, it is only possible to use interdependences between the form of the effectively transformed fold and its supporting conditions.

## 7. Conclusions

The presented dependencies describing the properties of the transformed folded sheets are very useful in the process of the rational shaping of attractive roof shell sheeting. They were derived on the basis of the analysis performed at various levels of accuracy. At the most detailed level related to single walls, flanges and webs of the transformed folds, the complex states of stresses appearing at particular points of these walls were analyzed. The highest values are achieved by the normal stresses acting transversely to the fold’s directions. These are tensile stresses acting in the flanges along the transverse ends of the shell folds. The values of these stresses are distributed in the range from 20 to 80 MPa for the unit twist angle *α_j_* = 4° and in the range of 40 to 120 MPa for *α_j_* = 5° depending on the type of the examined fold’s profiles.

The results of the performed analysis are consistent with the visible shape changes observed for adjacent folds in each transformed sheeting at the experimental stand. The longitudinal axes of two adjacent folds are mutual skew straight lines, so they approach each other halfway along their length, and move away from each other if they approach the transverse ends. Thus, the crosswise fold’s ends tend to expand. The compress stresses appear at a half along the length of each transformed fold, and the tensile stresses occur at the crosswise ends of the fold.

The presented analysis shows the usefulness of each transformed fold as a structural element, the mechanical properties of which are only slightly deteriorated by the initial transformations if the control and reduction of the level of the fold’s effort is applied in the manner proposed in this article. In this case, despite the large transformations of all folds and deformations of their walls, it is possible to obtain small values of the strains and the linear constitutive relationships resulting from the open profiles, small transverse bending stiffness and small torsional stiffness of the shell folds.

The conditions leading to the reduction of the initial stresses of each fold in a transformed shell are imposed in the analysis carried out at the second level of detail, lower than the previously mentioned one. At this level, smooth models of all single folds are shaped in the form of regular ruled surfaces whose properties preserve the relationships obtained on the basis of the experimental tests and computer simulations. The variable width increments appearing along the length of each transformed fold are modeled by means of a central sector of a smooth hyperbolic paraboloid characterized by (a) straight longitudinal edges, (b) variable width along its length, and (c) a straight line of striction perpendicular to these longitudinal edges and passing through the halves of all shell folds.

The limitations reducing the effort of the transformed fold are imposed by means of the appropriate position of its contracting line and the appropriate value of its surface area calculated in the process of shaping a transformed folded shell. The conducted analysis shows strongly non-linear relationships appearing between the essential geometric properties of the transformed shell sheeting and the degree of twist of each of its fold. These relationships significantly depend on the profile type of the examined folds.

A central sector of a right hyperbolic paraboloid is used as a simplified model of each fold after its transformation. In order to describe the relationships appearing between many specific properties of the shell fold in its various configurations, surface areas of the hyperbolic paraboloid models are used.

If we know the variation of the surface areas of the successive fold’s configurations, we are able to calculate the widths of the transformed fold in its subsequent configurations and the length of the contraction line of the entire transformed shell. Then, we can calculate the positions of the lines modeling the longitudinal and transverse edges of each shell fold and its state of stresses using the geometric properties of the central sector of a right hyperbolic paraboloid. These interdependences are going to be analyzed in the further publications. Based on the obtained relationships and smooth hyperbolic paraboloid models, it is possible to obtain displacement equations of all points of the entire transformed folded shell. The derivation of these equations is beyond the scope of the present article.

## Data Availability

Data Sharing is not applicable.
